# The Belowground–Aboveground Interactions of Zucchini: The Effects of *Trichoderma afroharzianum* Strain T22 on the Population and Behavior of the Aphid *Aphis gossypii* Glover and Its Endoparasitoid *Aphidius colemani* Viereck

**DOI:** 10.3390/insects15090690

**Published:** 2024-09-12

**Authors:** Donatella Battaglia, Stefania Mirela Mang, Vittoria Caccavo, Paolo Fanti, Pierluigi Forlano

**Affiliations:** 1 Department of Agricultural, Forestry, Food and Environmental Sciences (DAFE), University of Basilicata, Viale dell’Ateneo Lucano 10, 85100 Potenza, Italy; donatella.battaglia@unibas.it (D.B.); stefania.mang@unibas.it (S.M.M.); vittoria.caccavo@unibas.it (V.C.); paolo.fanti@unibas.it (P.F.); 2 Department of Pharmacy, University of Salerno, Via Giovanni Paolo II 132, 84084 Fisciano, Italy

**Keywords:** *Trichoderma harzianum*, dual-choice test, insect attraction, braconid wasp, mummification rate, parasitism rate, biological control

## Abstract

**Simple Summary:**

The use of benefic fungal species acting as plant growth promoters and defense inducers has been increasing in the last decades. *Trichoderma* fungi are among the most appreciated benefic fungi in agriculture. They colonize plant roots and activate systemic plant defenses against pests. *Trichoderma* may influence the physiology of the colonized plants; as a consequence, the behavior and the development of insects feeding on them may be affected. The main purpose of this present study was to investigate the effects of the inoculation of *Trichoderma afroharzianum* T22, a commercial fungal strain, on the population growth and behavior of the aphid *Aphis gossypii* and on the parasitism of its natural enemy *Aphidius colemani,* using zucchini as the model plant species. Our results show that the inoculation of *T. afroharzianum* T22 influences the behavior and the population growth of the aphid and its endoparasitoid. These results are discussed with regards to strategies for sustainable pest management.

**Abstract:**

Fungi belonging to the genus *Trichoderma* have received high consideration in agriculture due to their beneficial effects on crops from their plant promotion effects and protection from disease. A role of *Trichoderma* fungi in triggering plant defense mechanisms against insect pests, either directly or by natural enemy attraction, has been proposed, even if the results in different studies are controversial. In this present study, using zucchini plants as a model species, we investigated the effects of *Trichoderma afroharzianum* strain T22 plant inoculation on the cotton aphid *Aphis gossypii* and its endoparasitoid *Aphidius colemani*. Our results showed that the inoculation with *T. afroharzianum* T22 promotes *A. gossypii* population growth and makes zucchini more attractive to the aphid. The higher abundance of aphids on *Trichoderma*-inoculated zucchini was compensated for by a higher presence of the mummies of *Aphidius colemani*. In this present study, we recorded a higher zucchini biomass, thereby confirming that *Trichoderma* can act as a plant growth inducer.

## 1. Introduction

The use of beneficial fungi is currently increasing in agriculture due to their multifaceted roles [1,2,3,4]. *Trichoderma afroharzianum* (Ascomycota, teleomorph: Hypocrea), a filamentous fungus widespread worldwide in different soil types [5], colonizes root surfaces and penetrates the root epidermis limited to the first or second cell layer without destroying plant tissues [6]. Root colonization may strongly influence plant physiology due to the extensive molecular communication between the fungus and plant [7,8].

*Trichoderma* fungi are well known as plant growth promoters [8]. This ability has been attributed to different physiological processes triggered by the *Trichoderma* colonization of plant tissues, such as hormone secretion and secondary metabolite synthesis [9,10,11,12], which can determine a general increase in the photosynthetic activity of the colonized plants [12]. 

Laboratory and field studies have showed that *Trichoderma* fungi can also influence the plant response against sap-feeder species by altering their plant defense pathways [13,14,15], but the final result may be very different, even with the same species, for the aphid *Macrosiphum euphorbiae* (Thomas) if the *Trichoderma* strain is different: it can be detrimental in some studies [14,15] and beneficial in other ones [16]; Battaglia et al., unpublished]. As a result, the final outcome of the *Trichoderma*–aphid interactions may be not only pest-specific but also related to the fungal strain–plant genotype combination [17]. Some studies show that *Trichoderma* species can also influence the pest–parasitoid interactions and their population dynamics. For example, Contreras-Cornejo et al. [18] found that the colonization of maize roots by *T. atroviride* results in an increase in the parasitism rate of *Spodoptera frugiperda* (Smith) (Lepidoptera: Noctuidae) by its parasitoid *Campoletis sonorensis* (Carlson) (Hymenoptera: Ichnumoumonidae). Plant attractiveness to the aphid endoparasitoid *Aphidius ervi* Haliday (Hymenoptera: Braconidae) is increased by both *T. longibrachiatum MK1* [16] and *T. afroharzianum* T22 [14] plant inoculation. In addition, in two of our previous field studies, we found that *T. afroharzianum* T22 root inoculation makes tomato and [19] zucchini plants [20] more attractive to several *Hymenopteran* parasitoids. 

The ability of *Trichoderma afroharzianum* to affect pests has been investigated under field conditions [19,20,21], where pest abundance is the outcome of several uncontrolled factors (abiotic factors, parasitism, predation, plant resistance induction, and the plant’s nutritional value) and could be differentially affected by *Trichoderma*. Laboratory studies allow the assessment of the impact of single factors and then the interpretation of the field results. 

*Aphis gossypii* is the main aphid pest of cucurbits; it is capable of considerably damaging infested plants, both because of its feeding activity and its ability to transmit more than 50 viruses [22]. In greenhouses and tunnels, *A. gossypii* infestation on cucurbits may be controlled by using the braconid endoparasitoid *Aphidius colemani* Viereck [23,24]. *Aphidius colemani* is an aphidophagous endoparasitoid whose development cycle includes an egg stage, three larval stages, and a pupal stage. The biological cycle concludes with the hatching of the adult mummified body of the host aphid [25]. 

In this present study, we hypothesize that *T. afroharzianum* T22 inoculation on zucchini plants (San Pasquale cultivar) may affect both the behavior (i.e., attraction) and population growth of *A. gossypii* and its parasitoid *A. colemani*. In particular, in the first experiment, we hypothesized that *T. afroharzianum* T22 may influence *A. gossypii* population growth and some of its life history traits; this was investigated under two different fluctuating thermal conditions. In a second experiment, we hypothesized that T22 inoculation on zucchini plants may vary the plant attractiveness to the endoparasitoid A. colemani, as well as the final parasitization rate of *A. gossypii* by *A. colemani*. We also hypothesized that *T. afroharzianum* T22 aphid colony dispersion in Trichoderma-inoculated plants. In a third experiment, we compared the plant attractiveness of *Trichoderma*-inoculated and uninoculated plants to two apterous morphs (i.e., with different colors) and a winged stage *A. gossypii*.

## 2. Materials and Methods

### 2.1. Plant and Aphid Rearing

Zucchini plants (*Cucurbita pepo* L., cultivar San Pasquale) were used in this study. To enhance germination, the seeds were kept in water for 3 h, then placed on a wet cotton disc in sterile Petri dishes and stocked at 21 ± 2 °C in darkness. Only the germinated seeds were sown in cylindrical plastic pots (18 cm diameter, 15 cm height, and 1.5 L volume) containing commercial soil (COMPO SANA^®^ Universal Potting Soil—at a pH (H_2_0) of 6.5 with an apparent density of 150 kg/m^3^ and a porosity of 90% *v*/*v*). All the zucchini plants used in this study were fertilized twice by providing them with two different doses of an ammonium nitrate solution (2 gr/L—26% nitrogen). In particular, 50 mL of this solution was provided on the 1st and 5th days from the cotyledon emergence. The plants were irrigated twice a week by providing them with 300 mL of water/pot. All the zucchini plants used in this study were reared in a glass greenhouse at 21 ± 2 °C, in 40–50% relative humidity, and under a 16:8 (D: L) photoperiod. 

The *Aphis gossypii* colony utilized in this study was obtained by collecting, during the month of August 2022, about 50 adult specimens from an infested melon (*Cucubis melo* L.) (Pignola, Basilicata Region, Southern Italy). Identification was carried out on some adult specimens by using the keys formulated by Blackman and Eastop [22], and, after that, we started an aphid mass-rearing culture, using young (2-week-old) zucchini plants to rear the *A. gossypii*. The plants used for the aphid mass-rearing culture were illuminated with white LED tubes (20 W, 130 lm/W) and full-spectrum LED tubes (36 W, 100 lm/W165).

### 2.2. Trichoderma Treatment and Root Colonization Assessment

The viability of the *T. afroharzianum* (syn. *T. harzianum*) [26] Rifai T22 strain KRL-AG2 (KOPPERT B.V., Berkel en Rodenrijs, the Netherlands), a commercial formulation, was evaluated (as described in Forlano et al. [20]) before starting the laboratory experiments. In particular, the viability of the commercial formulation of *T. afroharzianum* T22 was evaluated in the laboratory by serial dilution. The dilutions were placed on Petri plates (9 cm in diameter) containing a potato dextrose agar (PDA) medium (Oxoid Ltd., Hants., UK), amended with 40 mg/L of streptomycin sulphate (MerckKGaA, Darmstadt, Germany), until growth could be detected. The Petri plates were incubated at 25 °C in the dark, and the number of colony forming units was counted after 24 h.

Once the viability of the product had been checked, the zucchini seedlings were inoculated. The root inoculation was performed twice on the 2nd and 5th days after germination. A spore suspension (1 gr/L; 1 × 10^9^ colony forming units/g of viable spores) of the commercial product containing *T. afroharzianum* was used. Specifically, 50 and 100 mL/pots of the spore suspension were provided for the first and second inoculations, respectively. For all the experiments of this study, the aphid infestation and behavioral tests were carried out by testing 16-day-old plants, which corresponded to the 14th day from the first fungal inoculation. To verify the zucchini root colonization by the *Trichoderma*, a fungus isolation on a potato dextrose agar (PDA) medium, supplemented with streptomycin sulfate (0.05%), was carried out, as described by Camele and Mang [27] and by Mang et al. [28]. At the end of each experiment, a random root collection from three *Trichoderma*-treated and three control plants was performed. The roots were carefully and singularly washed with sterile water to remove any soil. They were cut into small pieces 1–1.5 cm long, immersed in a 70% hydroalcoholic solution for 20 s, then in a 2% sodium hypochlorite solution for 20 s, and finally washed three times with sterile distilled water, before being paper-dried on sterile paper sheets. In addition, the H_2_O of the last rinse was also plated on the PDA (9 mm Ø plates) to verify the effectiveness of the sterilization technique [29]. Subsequently, three to four small sterilized roots were placed in Petri plates containing PDA amended with antibiotics, as described by Mang et al. [28], and incubated for 8 days at 24 ± 0.5 °C until fungal growth became visible. The presence of *T. afroharzianum* T22 was visually determined based on the morphological traits described by Gams and Bisset [30] and by Chaverri et al. [26].

### 2.3. Effects of Trichoderma on Aphid Life History Traits and Population Growth

The effects of *Trichoderma* on *A. gossypii* population growth and some of their main life history traits were investigated by using the first instar nymphs. In this experiment, the plants (that were 16 days old) were tested on the 14th day from the first *Trichoderma* inoculation. The control treatment consisted of coetaneous zucchini plants that experienced the same conditions but were not inoculated with *Trichoderma*. Ten coetaneous first instar nymphs/plant were tested. The nymphs were obtained by adult females of the dark morph (DM), which was the most abundant adult morph in our aphid rearing under the above-described conditions. The other two morphs were the yellow dwarf morph (YDM) and dark-winged morph (DWM). The adults were taken from the rearing colony by using a soft paintbrush and were subsequently put on a 2-week-old potted zucchini plant. The plant was then stocked in a meshed cubic plastic box (40 cm × 40 cm × 40 cm) under the same climatic conditions at which the aphid mass-rearing culture was maintained. After 3 h, the adult aphids were removed from the plant and the nymphs were used to infest the experimental plants. The nymphs used to infest the plants were randomly assigned to the plant. In particular, 2 or 3 nymphs/plant were put inside a clip cage (of 1.5 cm height and 1.5 cm diameter) and monitored to assess the effects of *Trichoderma* on their developmental times. The developmental time was estimated by considering the last molt and the occurrence of the adult stage. The effects of *Trichoderma* on the aphids’ pre-reproductive mortality and subsequent fertility were estimated by considering both the aphids inside the clip cages and those feeding on the plants. The fertility and aphid mortality at the adult stage were also checked during the first five days of the adult stage. 

The growth of *Trichoderma* and its capability to induce defenses in plants against insect pests are influenced by temperature [31], so we carried out this experiment under two different fluctuating thermal conditions, which both fall in its vital thermal range. In particular, this experiment was carried out under two different thermal conditions: at 25 ± 1 °C during the light hours and 21 ± 1 °C during the dark hours (condition T1) and at 20 ± 1 °C during the light hours and 17 ± 1 °C during the dark hours (condition T2). Three repetitions were carried out for each thermal condition. Each repetition consisted of five T22-inoculated and five control plants. The aphid population growth was observed for 13 days after the nymph infestation on the plants. 

### 2.4. Effects of Trichoderma on Parasitoid Attraction and Parasitism Rates

In this experiment, the effect of the *T. afroharzianum* T22 on *A. colemani* parasitism was evaluated by setting up an experimental arena hosting *Trichoderma*-treated plants and untreated plants, either infested with *A. gossypii* or not. The production of infested plants for the choice test was used as an opportunity to further verify the effect of *Trichoderma* on aphid mortality and population growth and to observe the dispersal behavior of the aphids on the plants.

#### 2.4.1. Aphid Population Growth and Colony Dispersion on Plants

The aphids used in this experiment were obtained from adult individuals of the DF. The adults were kept on young zucchini plants, in groups of 70 individuals per plant, for 8 h. The nymphs laid in this period were transferred onto the plants and kept until they reached adulthood (i.e., until they were 8 days old). The plant infestation was made by gently transferring, with a soft brush, twenty aphids to each plant. To investigate whether *Trichoderma* might have any dispersal effect on the aphids, all of the twenty adult aphids were released on the adaxial part of the second oldest leaf, which was considered as the plant starting point. The aphid population growth was checked on the 1st, 3rd, 5th, and 7th days after infestation by counting both the adult aphids and progeny. The mortality of the adult aphids was recorded on the 1st and 5th days from the beginning of the infestation. During the aphid counting, the number of aphids found on the starting point and the number of those found on the other plant parts were recorded. On the 7th day after the initial infestation, after the last aphid counting on the plants, the infested plants were used to set up the experimental arena to evaluate the possible effects of *Trichoderma* on *A. colemani*. In this experiment, too, the plants were used on the 14th day after the first *Trichoderma* inoculation.

#### 2.4.2. Parasitoid Choice Test

To investigate the effects of *Trichoderma* on zucchini plant attractiveness to the parasitoid *A. colemani*, a choice test was carried out by using *Trichoderma*-inoculated plants and control plants both infested with *A. gossypii*. This experiment was carried out in a greenhouse, and it was repeated three times, with 3 or 4 replicates/repetition (for a total of 11 replicates). Each replicate consisted of two groups of plants: one group composed of ten *Trichoderma*-inoculated plants and the other group composed of ten uninoculated plants. In each group, two plants were infested with *A. gossypii* and the other eight were not infested. In addition, each arena consisted of a table 4 m long and 1 m wide. The two plant groups were separately placed on the table to form two distinct plots, with each plot being 2 m long and 1 m large; as a result, each plot was half of the table surface. For both plots, the ten experimental plants were placed in two parallel rows, with five plants/row, and the two infested plants were placed in the middle part of the two rows. At the release of the parasitoids, the plants were 24 days old, which corresponded to the 21st day from the first *Trichoderma* treatment and to the 7th day from the beginning of the infestation. 

**Figure 1:**
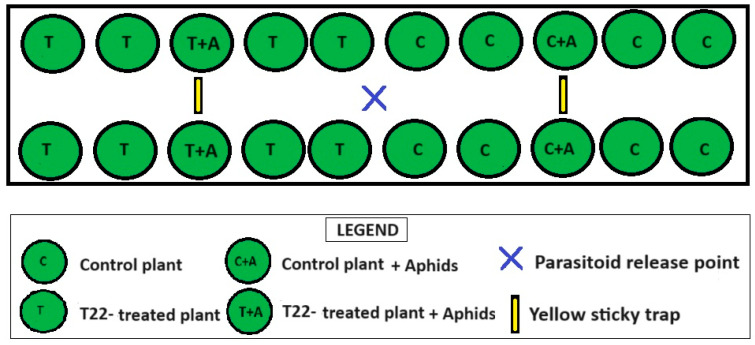
Experimental arena in which the parasitoid behavioral test was carried out.

The parasitoids used in this study were obtained as aphid mummies from Koppert Biological Systems Inc. (Howell, MI, Italy). At the arrival of the shipment from Koppert, the parasitoids that had already emerged were all discarded, because they were considered stressed due to starvation and disturbances during travel. To allow the parasitoids to experience the host-infested plant volatiles and enhance their olfactory response during the behavioral test [32], the mummies were put in a plastic cage (40 cm × 40 cm × 40 cm) containing a young zucchini plant infested with 50–60 *A. gossypii* adults. Before the parasitoid release in the experimental arena, in order to minimize any starvation effect, the parasitoids were restored by providing them with a 10% water–honey solution [33]. This solution was provided through a 15 mL Eppendorf tube, plugged with hydrophilic cotton to allow a constant food uptake by the parasitoids. Twenty-four hours after the parasitoid emergence, the adults were collected with a mouth aspirator and were singularly observed with a stereomicroscope to distinguish the females. The females and males were distinguished by considering the abdomen shape: it is rounded in males and pointed in females. The males were discarded and the females were enclosed in 50 mL Falcon tubes to form groups of 50 individuals that were at an equidistant position between the two different experimental plot types (Figure 1). To test the possible effects of *Trichoderma* on plant attractiveness, we recorded the movement of the *A. colemani* to the *Trichoderma*-inoculated plants or the control ones by using yellow sticky traps (10 cm × 25 cm) placed between the plants, as shown in Figure 1.

The number of parasitoids caught on the traps was counted on the 1st, 4th, and 24th hours after the parasitoid release. Twenty-four hours after the parasitoid release, the aphid-infested plants were accurately observed to remove the parasitoids and then stocked in a greenhouse cabin. Starting from the 5th day after the parasitoid release, the aphid colonies were observed daily to record the beginning of the aphid mummification. Twenty-four hours after the appearance of the first mummies (at least 2 mummies/plant), the number of mummies was counted for all the plants. The counting of the mummies was checked by observing the aphid colony under a stereomicroscope. The difference in the aphid mummy abundance between the *Trichoderma*-treated plants and the control ones was calculated by considering the total number of mummies found for the two experimental plant types. The parasitism rates were calculated by considering the number of aphids per plant on the 7th day from the plant infestation, which was the day of the parasitoid release and the number of mummified aphids found after 24 h from the beginning of the aphid mummification.

### 2.5. Effect of Trichoderma on Aphid Attraction 

#### 2.5.1. Dual-Choice Test with Apterous Aphids

The effect of *Trichoderma* on apterous aphid attraction was tested through double-choice assays. In particular, a group of coetaneous adult aphids was allowed to choose between a *Trichoderma*-inoculated zucchini plant or a coetaneous non-inoculated plant. The effects of the *Trichoderma* on aphid attraction were separately tested for both the dark morph (DM) and the yellow dwarf morph (YDM) of *A. gossypii*. Fifteen adult individuals of the DM (that were 8 days old) and ten individuals of the YDM (that were 7 days old) were separately tested using a couple of plants. The two experimental plants were connected through a cardboard bridge (20 cm x 4 cm). In particular, the bridge was placed to connect the second older leaf of the two tested plants. The aphids were subjected to a starvation period of 1 h, a time period considered appropriate in similar studies on aphids [34]. During the starvation period, the aphids were stocked in 15 mL Eppendorf tubes at room temperature (20 ± 1 °C). The choice test was started by the aphid release at the middle of the bridge. After 1 h, the aphids were located on the plants to investigate any preferences between the inoculated and uninoculated plant. 

This experiment was carried out in laboratory-controlled climatic conditions (20 ± 1 °C, 55 ± 10% RH). All the tested plants used in this experiment were 16 days old, which corresponded to the 14th day from the first *Trichoderma* inoculation. Three repetitions, with nine replicates/repetition, were carried out (twenty-seven replicates) for the DF. In the case of the YDF, twelve repetitions, consisting of 2–3 replicates/repetitions, were carried out (30 replicates). 

#### 2.5.2. Dual-Choice Test with the Winged Aphid

The effects of *Trichoderma* on plant attractiveness to *A. gossypii* were also tested on the dark-winged aphid (DWA). For this experiment, the arena consisted of a mesh-covered cage (with a base that was 60 cm x 60 cm; 100 cm height) with a platform on the top to allow the positioning of the aphids above the tested plants. Two *Trichoderma*-inoculated and two uninoculated zucchini plants were used for each replicate. Each of the four plants was positioned at a corner of the cage base, alternating the *Trichoderma*-treated plants and the untreated ones. All the DWM specimens used in this study were collected by the mass-rearing culture when at the 3rd and 4th nymphal stage, chosen by considering the presence of wingpads. After that, the collected nymphs were kept on the small zucchini plants and placed within a mesh-covered cage. The behavioral tests were carried out on the adults, and just the specimens that reached the top of the cage were used, because they were considered mature and capable of flying. Forty specimens for each replicate were simultaneously tested. Each test lasted 24 h. At the end of the test, the number of DWAs was counted in each of the four plants, as well as the number of nymphs deposed by the DWAs on the plants. Due to the very small size of the progeny, the nymphs were counted by observing the zucchini shoots under a stereomicroscope. 

All the tested plants were 16 days old; this experiment was carried out under the same conditions as described above for the dual-choice test concerning the apterous aphids. Fifteen independent replicates were set up.

### 2.6. Effect of Trichoderma on Plant Biomass

To investigate whether *T. afroharzianum T22* plays a role as a plant growth inducer on zucchini plants, the fresh and dry weights of the roots and shoots were measured at the end of the experiment, in which we tested the effect of *Trichoderma* on the parasitism of *A. colemani* (using 30-day-old plants). The zucchini plant parts and tissues were placed in an oven (Hotbox oven, Gallenkamp, UK, size 2) at 85 °C for 24 h to obtain the dry weight. Sixteen plants were subjected to these measurements for each of the two experimental treatments. 

### 2.7. Statistical Analysis 

In Experiment 1, the colony growth over time was analyzed using a two-way factorial ANOVA with “treatment” and “day” as the main fixed effects. In the case of the plant infestation, starting with the nymphs, nine levels (from day 0 to day 13) were present. In the experiment in which the plant infestation was started by the adults, four levels (from day 0 to 6) were present. For these data, the assumptions of homoscedasticity and normality for the ANOVA were checked. In the colony growth experiments, the data on developmental times, survival, and fertility were normally distributed, and two-sample *t*-tests for the differences between the treatments were applied. In the case of fertility, a Log transformation of the data was performed.

To test whether the inoculation with *T. afroharzianum* affected the aphid dispersal on the zucchini plants (Exp. 2), the number of aphids found in a different position from the leaf on which they were initially placed was recorded and standardized to the total number of individuals on the plant on a given day. Furthermore, in this case, the dispersal over time was analyzed using a two-way factorial ANOVA with “treatment” and “day” (with 4 levels: from day 0 to day 6) as the main fixed effects. The homoscedasticity and normality assumptions for the ANOVA were checked. 

In the case of the behavioral tests, both for aphids and parasitoids, the raw data used in the plant preference analyses were the percentages of the specimens found on either the *Trichoderma*-treated or the control plants. The effect of *Trichoderma* on *A. colemani* parasitism was evaluated by considering the total number of mummies found on the four infested plants constituting each replicate and the percentages of mummies found on the *Trichoderma*-treated and untreated plants, which were calculated by considering the total number of mummies. For the behavioral tests, the preference of aphids to colonize the two plant types was evaluated by considering the total number of individuals found on the plants constituting each replicate and the percentages of aphids found on the *Trichoderma*-treated and untreated plants were calculated by considering the total number of individuals. The homoscedasticity and normality assumptions were tested and met for these data. The aphid preference between the *Trichoderma*-treated and untreated plants was evaluated by considering the mean percentage of the individual preference ±95% confidence intervals; these values were compared to the value of 50%. The equality of the percentages of the individual preferences between the two plant types of 50% was in accordance with the null hypothesis that there is no difference in insect preference between the *Trichoderma*-treated and control zucchini plants. The differences in the parasitism rates were analyzed using a one-way ANOVA.

The differences in the fresh and dry weights of the zucchini shoots and roots between the *Trichoderma*-treated and control plants were analyzed using a two-sample *t*-test. 

## 3. Results 

### 3.1. Trichoderma Inoculation and Colonization

The sterilization procedure was effective, because no fungal colonies were found after 1 week in the Petri plates in which the water of the last rinse was inoculated. As found in our previous studies [19,20], in which the same commercial formulation was used and similar inoculation protocols were adopted, the presence of T22 was observed in all the treated plants, while the Petri dishes containing the root parts of the untreated plants were free of *T. afroharzianum* T22.

### 3.2. Effects of Trichoderma on Aphid Life History Traits and Population Growth

#### 3.2.1. Developmental Time, Immature and Adult Survival, and Fertility

The developmental times, under the T1 thermal condition (25–21 °C), were 142.25 (±0.62) hours in the case of the aphid developed on the *Trichoderma*-treated plants and 143.86 (±0.50) hours in the case of the aphids developed on the control plants. Under the T2 thermal condition (20–17 °C), the developmental times were 164.16 (±0.54) hours on the *Trichoderma*-treated plants and 165.00 (±0.59) hours on the control plants. A statistical analysis showed no significant differences in the developmental time (T1: t_27_ = 0.21, *p* = 0.95; T2: t_24_ = 0.12, *p* = 0.95). 

The survival during the immature period under the T1 thermal condition (25–21 °C) was 81.33% (±2.91) in the case of the aphids developed on the *Trichoderma*-treated plants and 78.0% (±3.35) in the case of the aphids developed on the control plants. Under the T2 thermal condition (20–17 °C), the survival of the immatures was 84.0% (±2.35) on the *Trichoderma*-treated plants and 82.67% (±2.70) on the control plants. No differences were found in the aphid survival during the immature period on the *Trichoderma*-treated plants and control plants for any of the two considered thermal conditions (T1: t_14_ = 0.47, *p* = 0.95; T2: t_14_ = 0.27, *p* = 0.95). 

The survival during the first five days of the adult stage under the T1 thermal condition (25–21 °C) was 80.67% (±2.28) in the case of the aphids developed on the *Trichoderma*-treated plants and 81.33% (±2.15) in the case of the aphids developed on the control plants. Under the T2 thermal condition (20–17 °C), the survival of the immatures was 78.67% (±2.15) on the *Trichoderma*-treated plants and 76.0% (±3.06) on the control plants. No difference in the survival between the aphid colonies of the *Trichoderma*-treated plants and the aphid colonies of the control plants was found during the first five days of the adult stage under both of the thermal conditions (T1: t_14_ = 0.41, *p* = 0.95; T2: t_14_ = 0.24, *p* = 0.95).

The fertility during the first five days of the adult stage under the T1 thermal condition (25–21 °C) was 45.36 (±1.95) nymphs/aphid in the case of the aphids developed on the *Trichoderma*-treated plants and 39.53 (±1.35) nymphs/aphid in the case of the aphids developed on the control plants. Under the T2 thermal condition (20–17 °C), the survival of the immatures was 21.71 (±0.6) nymphs/aphid on the *Trichoderma*-treated plants and 18.86 (±0.71) nymphs/aphid on the control plants. The fertility was higher on the *Trichoderma*-treated plants than on the control plants under both the investigated thermal conditions. A statistical analysis confirmed significant differences between the two experimental treatments (*p* < 0.05).

#### 3.2.2. Aphid Population Growth

**Figure 2:**
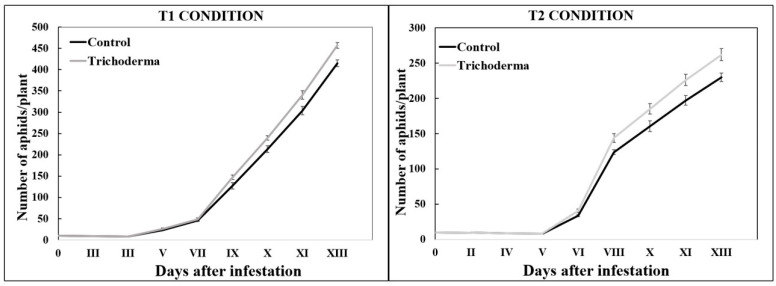
Development of the aphid colony on *Trichoderma*-treated and untreated plants. Mean values (±SE) of the number of live *A. gossypii* counted over time under T1 and T2 thermal conditions.

On the 14th day after infestation, under the T1 conditions, the mean number of *A. gossypii* per plant was 456.93 (±6.5) on the *Trichoderma*-treated plants and 415.07 (±8.3) on the control plants; under the T2 conditions, the mean number of *A. gossypii* was 261.8 (±8.5) on the *Trichoderma*-treated plants and 229.60 (±6.0) on the control plants (Figure 2). In the *Trichoderma*-inoculated plants, with respect to the control ones, we checked a greater aphid population abundance of 9.17% (under the T1 condition) and of 12.29% (under the T2 condition). A statistical analysis showed significant differences in the aphid population abundance between the two considered plant treatments, both under the T1 (F_3.878_ = 71.453, *p* < 0.001) and the T2 (F_3.878_ = 32.070, *p* < 0.001) conditions, confirming that *T. afroharzianum* T22 significantly promotes *A. gossypii* population growth on zucchini plants. 

### 3.3. Effects of Trichoderma on Aphid Population and Dispersal and on Parasitoid Attraction and Parasitism Rates

#### 3.3.1. Aphid Population and Life History Traits

On the first day, the aphid survival was 86.59% (±1.66) on the *Trichoderma*-treated plants and 85.91% (±1.66) on the control plants. On the fifth day, the aphid survival was 80.91% (±0.71) on the *Trichoderma*-treated plants and 79.55% (±1.50) on the control plants. A statistical analysis showed no significant differences, indicating that *T. afroharzianum* did not affect the survival of *A. gossypii* adults on zucchini plants during the first week after infestation. 

**Figure 3:**
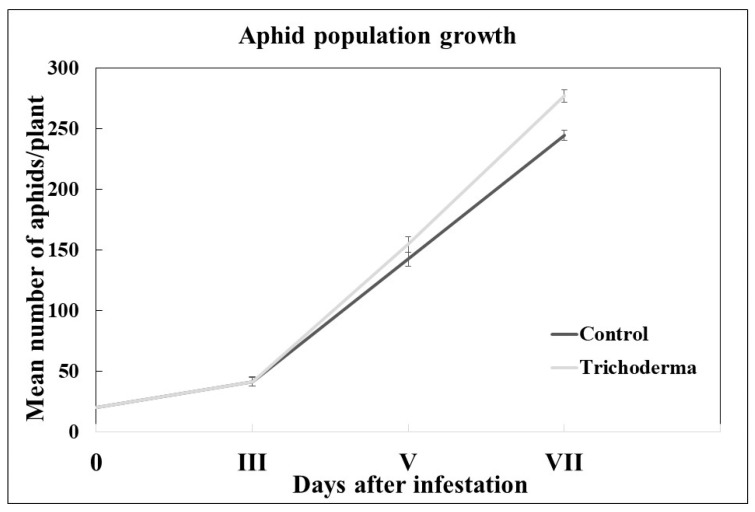
Development of the aphid colony on *T. afroharzianum*-treated and control zucchini plants. Mean values (±SE) of the number of live *A. gossypii* over time.

On the seventh day from the beginning of the infestation, the mean number of aphids per plant was higher on the *Trichoderma*-treated plants than on the control plants. In particular, the infestation consisted of 277.09 (±5.25) aphids on the *Trichoderma*-treated plants and 244.50 (±3.93) aphids on the control plants (Figure 3). Statistical analyses showed significant differences in the aphid abundance on the *Trichoderma*-treated vs. control plants (F_1,897_ = 13.89 *p* < 0.001), confirming that *T. afroharzianum* T22 inoculation on zucchini plants promotes *A. gossypii* population growth. Furthermore, *T. afroharzianum* determined an aphid population increment of 11.76% when compared to the aphid population on the control plants.

#### 3.3.2. Dispersal Effect of Trichoderma on the Aphid Colony

**Figure 4:**
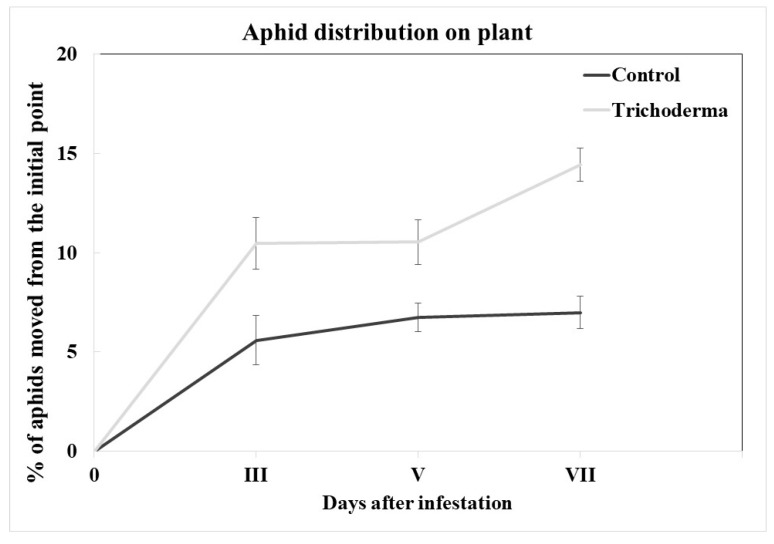
Mean values (±SE) of the percentage of the *A. gossypii* individuals recorded over time on plant leaves other than the initial one, on the control plants or the *T. afroharzianum*-treated ones.

On the seventh day after the initial plant infestation, on the *Trichoderma*-treated plants, 85.41% of the aphids were found on the initial point, while the remaining 14.59% of the colony was found on the other plant parts. On the control plants, 93.20% of the colony was found on the initial point, and the remaining 6.80% was found on the other plant parts (Figure 4). A statistical analysis showed a difference in the aphid dispersion of *A. gossypii* on the inoculated plants vs. the uninoculated ones (F_3,897_= 32.851, *p* < 0.001), confirming that the *Trichoderma* treatment induces a significantly higher aphid dispersal on these plants compared to the control plants.

#### 3.3.3. Parasitoid Choice Assay

**Figure 5:**
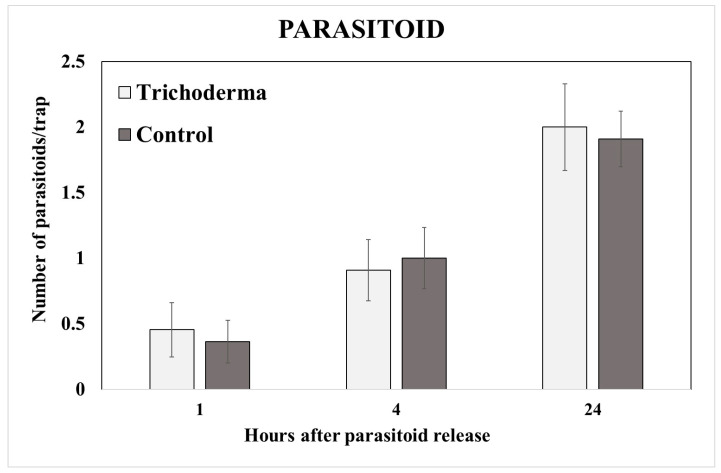
Yellow sticky trap capture. Mean number (±SE) of *A. colemani* individuals caught on yellow sticky traps on the 1st, 4th, and 24th hours after the parasitoid release.

After the parasitoid release, the number of parasitoids caught on the yellow sticky traps was checked on the 1st, 4th, and 24th hours. Statistical analyses showed no differences in terms of the parasitoids caught on the sticky traps positioned between the treatments (F_4,001_, *p* = 0.87) in none of the three considered times during the first 24 h after the parasitoid release (Figure 5). 

**Figure 6:**
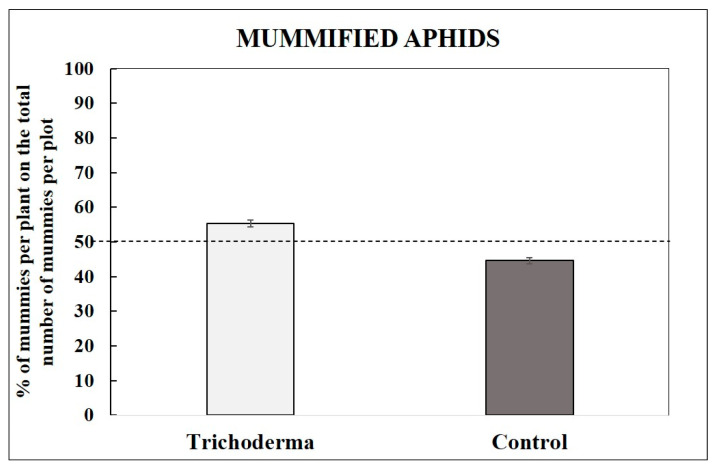
Mummification on the two plant types. Mean number (±SE) of the percentage of mummified aphids found on the *Trichoderma*-treated plants and on the control ones, considering the total number of mummies/plot.

On the *Trichoderma*-treated plants, we recorded 55.34% (±0.89) of mummies considering the total amount of mummies in both treatments (Figure 6). A statistical analysis showed differences between the two treatments (F_4.351_, *p* < 0.01).

The mean parasitism rate on the *Trichoderma*-treated plants was 8.94% (±0.43) and it was 8.23% (±0.48) on the control plants. A statistical analysis showed no differences in terms of the parasitism rate between the aphid colonies on the *Trichoderma*-treated plants and those on the control plants (F_4.061_, *p* = 0.30).

### 3.4. Effect of Trichoderma on Aphid Attraction

#### 3.4.1. Attraction of the Apterous Aphids

**Figure 7:**
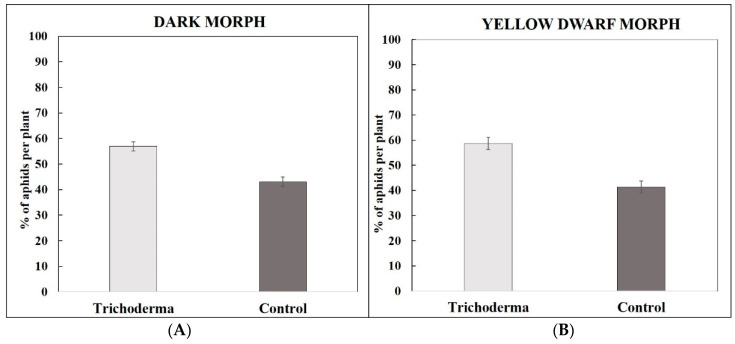
Dual-choice test of *A. gossypii* apterous aphids. Mean percentages (±95% confidence intervals) of the “dark morph” (**A**) and the “yellow dwarf morph” (**B**) of *Aphis gossypii* found on *T. afroharzianum*-treated and control zucchini plants.

*Aphis gossypii* preferentially colonized the *Trichoderma*-treated zucchini plants rather than the control plants under a choice condition. In the case of the DF, 56.93% (±1.79) of the aphids colonized the *Trichoderma*-treated plants (Figure 7A). In the case of the YDF, 58.67 (±2.43) of the aphids colonized the *Trichoderma*-treated plants and 41.33% (±2.43) colonized the control ones (Figure 7B). A statistical analysis showed differences between the treatments both in the case of the DF (F_4.026_, *p* < 0.01) and the YDF (F_4.006_, *p* < 0.01).

#### 3.4.2. Attraction of the Winged Morphs

**Figure 8:**
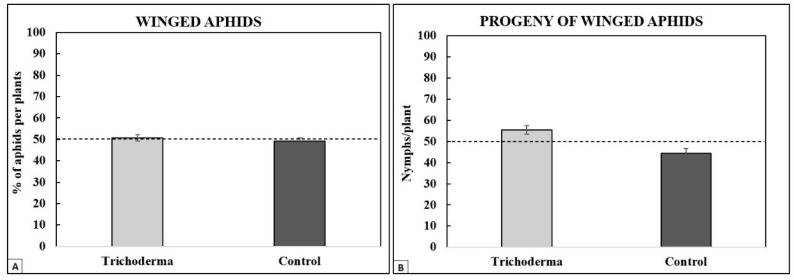
Dual-choice test with winged aphids. Mean percentages (±95% confidence intervals) of winged *A. gossypii* (**A**) and its progeny (**B**) found on *T. afroharzianum*-treated or control zucchini plants.

Twenty-four hours after the aphid release in the experimental arena, 50.75% (±1.44) of the individuals were found on the *Trichoderma*-treated plants. A statistical analysis showed that there was no significant difference in terms of preference for the WF of *A. gossypii* between the *Trichoderma*-treated and control plants (Figure 8A). On the *Trichoderma*-treated plants, 55.47% (±2.02) of the progeny was found, and the remaining 44.53% (±2.02) was found on the control plants (Figure 8B). The observed mean number of nymphs counted on the *Trichoderma*-treated zucchini was significantly higher than their respective theoretical means (i.e., 50–50%, the null hypothesis), indicating that the winged aphids preferentially deposed nymphs on the *Trichoderma*-treated zucchini rather than on the untreated ones.

### 3.5. Zucchini Plant Growth

**Figure 9:**
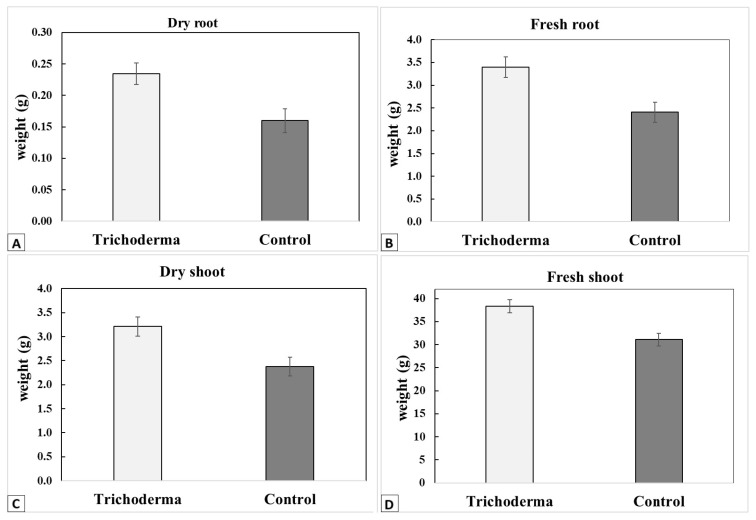
Zucchini plants’ biomass. Mean values (±SE) of the dry (**A**) and fresh (**B**) weights of the roots and the dry (**C**) and fresh (**D**) weights of the shoots for the *Trichoderma*-treated and control zucchini plants.

An increase in biomass was found in the *Trichoderma*-treated zucchini plants when the fresh (root: t_15_ = 0.001; shoot: t_15_ = 0.003) and dry (root: t_15_ = 0.003; shoot: t_15_ = 0.0003) weights were measured (Figure 9) on 30-day-old zucchini plants. 

## 4. Discussion

*Trichoderma*, like other root-associated soil microorganisms, is particularly appreciated in agriculture for its role in promoting plant growth [8,35] and activating plant defenses against abiotic [36] and biotic stresses [13,37]. In general, the effects of *Trichoderma* on the plant and population growth of phytophagous insects may be very variable. These effects are both influenced by plant nutritional value variations and the inducible defense activation, which may be considered two effects leading to conflicting results [38].

In our study, the *A. gossypii* population growth was faster on *Trichoderma*-treated plants than on untreated ones. This finding is in accordance with that of Battaglia et al. [16], in which *M. euphorbiae* showed a substantial population growth increment feeding on tomato plants colonized by *T. longibrachiatum* MK1. We can assume that, in both cases, the better aphid performance is due to the higher nutrient value of the *Trichoderma*-treated plants. The biomass increment found in the *Trichoderma*-treated zucchini plants supports this hypothesis and confirms the ability of this fungus to act as a plant growth inducer [8]. Probably, the increase in the zucchini biomass may also correspond to a rise in the plant nutritional value for aphids. In fact, *Trichoderma* fungi generally improve the nitrogen use efficiency in plants [39,40], therefore determining an augment in the total nitrogen content in the colonized plant [21]. It is well known that a higher content of nitrogen in plants increases both growth and fitness in aphids [41,42].

In our study, *A. gossypii* had a higher fertility when feeding on *T. afroharzianum* T22-treated plants than on untreated ones, as already reported by Trotta et al. [36] for *M. euphorbiae* on tomato plants inoculated with the same *Trichoderma* strain. Contrary to what has been reported by Trotta et al. [36] for *M. euphorbiae* on tomato plants, we did not find any differences in terms of aphid survival between *Trichoderma*-treated and untreated plants, neither during the immature stages nor during the first five days of adulthood in either of the two different experimental thermal conditions. This different observation could be due to a higher induction of the physiological responses triggered in *Trichoderma*-treated tomato plants than *Trichoderma*-treated zucchini plants, as well as a different response in the two different aphid species. 

The aphid colony dispersal on zucchini plants was higher on the *Trichoderma*-treated plants than on the uninoculated ones. It is known that *Trichoderma* root colonization in plants triggers the activation of the salicylic acid pathway, and, as a result, this can change the attractiveness of plants to insects [16]. The salicylic acid pathway is also inducible by sap-feeder insects, like aphids, due to the direct damage to plant tissues through their feeding [43,44]. It is known that methyl salicylate production and the VOC synthetized during the activation of the salicylic acid pathway affect the aphids’ behavior on plants [45,46]. In addition, methyl salicylate synthesis consistently increases over time in response to aphid infestation [47]. As demonstrated by Coppola et al. [45], in zucchini plants, the *A. gossypii* feeding activity activates the salicylic acid pathway according to the stress intensity due to aphid feeding. This activation, in turn, affects the aphid colony dispersal on zucchini plants. The higher dispersion of *A. gossypii* on the *Trichoderma*-treated plants might be due to a variation in the VOC’s synthesis, which could be varied both for the fungal colonization and the higher aphid population abundance. Then, both a direct and indirect effect of *Trichoderma* on aphid colony dispersion on zucchini plants could have occurred. 

The behavioral tests showed that *T. afroharzianum T22* increases the attractiveness of zucchini plants to apterous *A. gossypii*, in accordance with our previous findings in the field [20]. The outcomes of this present study suggest that *Trichoderma* inoculation is sufficient to induce variations in young zucchini plants (that are 16 days old), making it more vulnerable to aphid colonization. The capability of *Trichoderma* fungi to increase plant attractiveness to parasitoids has been documented in different studies [14,16,19,20,21]. Caccavo et al. [19] found a higher number of parasitoids on yellow sticky traps in *T. afroharzianum T22*-treated tomato plots than on the untreated ones, as also demonstrated by Coppola et al. [14] in the case of *Aphidius ervi* on tomato plants. Battaglia et al. [16], under laboratory conditions, found that tomato root colonization by *T. longibrachiatum* MK1 resulted in a higher attractiveness, in terms of oriented flights, to the braconid parasitoid *Aphidius ervi*. Contreras-Cornejo et al. [21] reported that root inoculation with *T. afroharzianum* increased the presence of ichneumonid parasitoids on maize foliage. Contrary to our previous field study [20], in which a higher attractiveness of *T. afroharzianum* T22-treated zucchini to hymenopteran parasitoids was demonstrated, in this study we found no differences in terms of the captured individuals through yellow sticky traps. This could be due to the different capture efficiencies of pan traps and sticky traps.

Differences between the treatments were found when the effects of *T. afroharzianum* T22 on *A. colemani* were evaluated considering the percentage of mummified aphids (as a proportion of the mummies per plant type concerning the total number of mummies found on both the plant types). In particular, the *A. colemani* mummies were more abundant on the *Trichoderma*-treated plants than on the control plants. On the contrary, when the effect of *T. afroharzianum* T22 on *A. colemani* parasitism was evaluated in terms of the mummification rate (number of mummies one day after the beginning of the mummification/number of aphids before the parasitoid release), differences between the inoculated and uninoculated plants were not found. Then, the higher aphid abundance on the *Trichoderma*-treated plants was compensated for by a higher number of mummies per plant, so there was no difference in terms of the parasitism rate between *Trichoderma*-treated and untreated zucchini plants. The parasitism of *A. colemani* on the aphid colony is host density-dependent, as documented by Jarošík and Lapchin [48]. The higher aphid abundance on *Trichoderma*-treated plants may have increased the plant attractiveness to the parasitoid. It is well known that the synthesis of VOCs during the expression of defensive pathways changes as a function of the physiological stress related to the different duration and intensity of the aphid infestation [47,49]. It is also possible that a biomass increase in Trichoderma-inoculated plants results in a greater VOC synthesis.

In conclusion, *T. afroharzianum* T22 inoculation via the root promotes the population growth of *A. gossypii* and makes zucchini more attractive to this pest. The higher aphid abundance on the *Trichoderma*-treated zucchini plants is compensated for by a higher number of mummies of *A. colemani*, which ensures an equal parasitism rate if compared to that of the untreated plants. Our findings support the hypothesis that the lower pest abundances found in *Trichoderma*-inoculated plants in the field [20] are principally due to the activity of natural enemies.

Although a great amount of information on the physiological interactions between *Trichoderma* fungi and plants is available [8,13], the outcomes of these interactions on insect pests and their natural enemies could be very variable. These complex and variable outcomes need to be investigated in multitrophic contests to support the development of suitable plant protection strategies. Our findings increase the knowledge of the effects of the cross-talk between *Trichoderma* and zucchini on the behavior and reproduction of an important aphid pest and on its main natural enemy.

## Data Availability

The data supporting the reported results can be made available upon request from the corresponding authors.
